# Ventilation heterogeneity across A-B-E phenotypes in COPD: insights from spirometry and electrical impedance tomography

**DOI:** 10.3389/fmed.2025.1731427

**Published:** 2025-11-27

**Authors:** Junsong Zhang, Tian Liu, Zhongkai Chang, Meng Dai, Liqiang Song, Lin Yang, Xinyu Ti, Shuoyao Qu, Zhanqi Zhao

**Affiliations:** 1 Department of Pulmonary and Critical Care Medicine, Xijing Hospital, Fourth Military Medical University, Xi’an, China; 2 Nanshan School, Guangzhou Medical University, Guangzhou, China; 3 The Sixth Clinical Medical School, Guangzhou Medical University, Qingyuan People’s Hospital, Guangzhou, China; 4 Department of Biomedical Engineering, The Fourth Military Medical University, Xi’an, China; 5 Department of Aerospace Medicine, The Fourth Military Medical University, Xi’an, China; 6 School of Biomedical Engineering, Guangzhou Medical University, Guangzhou, China

**Keywords:** electrical impedance tomography, chronic obstructive pulmonary disease, ABE phenotype classification, multiclass logistic regression model, regional ventilation

## Abstract

**Purpose:**

This study aimed to evaluate the regional ventilation distributions in A-B-E phenotypes among patients with chronic obstructive pulmonary disease (COPD). The feasibility to better distinguish the phenotypes combining global spirometry and regional ventilation parameters derived from electrical impedance tomography (EIT) was explored.

**Methods:**

A cohort undergoing pulmonary function testing was prospectively enrolled. Regional spatial and temporal ventilation parameters were calculated with EIT. Principal component analysis was used to visualize phenotypic clustering, while multinomial logistic regression evaluated discriminatory performance. Feature importance was interpreted using SHapley Additive exPlanations (SHAP).

**Results:**

This study enrolled 88 COPD patients (Group A *n* = 36, Group B *n* = 21, Group E *n* = 31). Spirometry and EIT parameters revealed significant intergroup differences in FEV_1_%pred (*P* < 0.001), FEV_1_/FVC (*P* < 0.001), GI-FEV_1_ (regional distribution of FEV_1_%pred in functional EIT; *P* = 0.004), GI-FEV_1_/FVC (regional distribution of FEV_1_/FVC; *P* = 0.001) and expiratory time constant (*P* = 0.017). Group A demonstrated the best pulmonary function (FEV_1_%pred: 77.67 ± 20.40), while Group E showed the most pronounced flow limitation (longest time required to exhale 75% of FVC, T75). The multinomial model showed optimal discrimination for Group A (AUC: 0.827), while differentiation between Groups B and E was less satisfactory (AUC: 0.749). SHAP analysis identified FEV_1_%pred as the most significant predictor (|SHAP| = 0.477), with EIT-derived parameters GI-FEV_1_/FVC (|SHAP| = 0.203) and regional T75 (|SHAP| = 0.189) providing substantial incremental value.

**Conclusion:**

COPD phenotypes showed differences in global and regional flow limitations. The combination of global and regional information helped with distinguishing phenotypes.

## 1 Introduction

Chronic obstructive pulmonary disease (COPD) is a highly prevalent condition associated with significant morbidity and mortality. According to data published by the World Health Organization in 2024, COPD was responsible for approximately 3.5 million deaths in 2021, accounting for about 5% of all global fatalities (1). The substantial disease burden of COPD stems primarily from its core pathological features: small airway disease (obstructive bronchiolitis) and parenchymal destruction (emphysema).

The “ABE” classification, one of the widely used tools in COPD management, stratifies patients into three groups—Group A (low risk of acute exacerbation, fewer respiratory symptoms), Group B (low risk, more symptoms), and Group E (high risk)—based on an integrated assessment of symptom burden and history of exacerbations (2). Given that patients in Groups A, B, and E differ markedly in terms of the degree of lung function impairment, symptom severity, and risk of exacerbations, tailored therapeutic strategies are required for each group. Among them, Group E, characterized by a high-risk profile, represents a critical subgroup in which timely identification and intervention could significantly slow disease progression, reduce the frequency of acute exacerbations, and improve overall patient outcomes (2).

Spirometry is considered the gold standard for diagnosing COPD and assessing the severity of airflow limitation. It also provides valuable information regarding the risk of exacerbations (2), and thus has certain sensitivity in distinguishing Group E patients (2, 3). However, current evidence indicates that relying solely on spirometry is insufficient for accurately differentiating among the ABE groups, particularly in discerning between Groups A and B, which exhibit a dissociation between symptoms and the degree of airflow obstruction (4). This underscores the need to incorporate additional diagnostic modalities to enhance patient stratification.

Electrical impedance tomography (EIT) is a non-invasive, real-time imaging technique that has gained increasing application in both experimental and clinical research (5). By providing regional information on ventilation distribution, EIT can effectively detect subclinical or occult pulmonary abnormalities (6), subtle pathological changes (7), and alterations in regional ventilation during acute exacerbations (8). It is regarded as a valuable adjunct to spirometry, offering complementary insights into regional lung function that are not captured by conventional spirometry (9). From a phenotypic perspective, patients in Group B often present with a chronic bronchitis-predominant phenotype (exacerbator with chronic bronchitis) (10, 11), whereas those in Group E are more likely to exhibit an emphysema-predominant phenotype (exacerbator with emphysema) (11, 12). Therefore, EIT-derived metrics may offer potential utility in distinguishing among the ABE groups.

This study aimed to evaluate the regional ventilation distributions in A-B-E phenotypes among patients with chronic obstructive pulmonary disease (COPD). The feasibility to better distinguish the phenotypes combining global spirometry and regional ventilation parameters derived from EIT was explored.

## 2 Materials and methods

### 2.1 Ethical approval and informed consent

This prospective study was approved by the Ethics Committee of the First Affiliated Hospital of Air Force Medical University (Approval No.: KY20242002-C-1). The research was conducted in accordance with the principles of the Declaration of Helsinki and local regulatory requirements (13). All participants provided written informed consent before enrollment.

### 2.2 Inclusion and exclusion criteria

Patients aged 40–80 years with a confirmed diagnosis of COPD, based on spirometry showing a post-bronchodilator FEV_1_/FVC ratio < 0.7 during a stable disease phase, were eligible for inclusion.

Exclusion criteria included any of the following: hospitalization requirement; presence of malignancy, autoimmune diseases, systemic infections, or major organ dysfunction; pleural disease or chest wall deformity; and contraindications to forced vital capacity (FVC) maneuver or EIT measurement, such as the presence of a pacemaker or implantable cardioverter-defibrillator.

### 2.3 Data collection

Participants first completed a basic demographic questionnaire, the COPD Assessment Test (CAT), and the modified Medical Research Council (mMRC) dyspnea scale. Subsequently, both spirometry and EIT assessments were performed concurrently. EIT measurements were conducted using the VenTom-200 EIT instrument (MidasMED Biomedical Technology, Suzhou, China). An electrode belt with 16 equidistant fixed electrodes was placed around the chest at the horizontal section corresponding to the 5th intercostal space on the parasternal line to capture regional ventilation distribution images. For female subjects, if the 5th intercostal space could not be reached, the electrode belt was placed above the breast (approximately at the 4th intercostal space) (14). Testing was conducted with participants in a seated position. The detailed information of the measurement process has been described in a previous study (9).

Pulmonary Function Test (PFT) was conducted using MasterScreen (Jaeger, CareFusion GmbH, Hoechberg, Germany). Following a standardized explanation of the FVC procedure, participants performed multiple efforts to achieve optimal and reproducible results, in compliance with the acceptability and repeatability criteria outlined in the 2019 American Thoracic Society (ATS) guidelines (15). Electrode contact impedance was monitored throughout the EIT procedure to ensure data quality.

### 2.4 Measured variables and classification model development

The functional EIT images, including FEV_1_ and FEV_1_/FVC maps, were calculated (16). The spatial heterogeneity of the functional images was assessed with the so-called GI index (17). Mean regional time to exhale 75% of FVC (T75) and time constant (τ) were calculated to evaluate the temporal heterogeneity (7, 16). In the present study, the time constant τ was referred to the product of the median and the interquartile range (IQR).

Additionally, FEV_1_% pred and FEV_1_/FVC were included as supplementary spirometric indices for comprehensive analysis.

All continuous variables, including both spirometry and EIT parameters, were standardized using Z-score normalization. A five-fold cross-validation framework was applied for data partitioning. Principal component analysis (PCA) was conducted on the standardized dataset for exploratory purposes, and the results were visualized to assess the natural separation of phenotypes (Groups A, B, and E) in reduced-dimensional space. A multinomial logistic regression model was developed with a three-level categorical outcome: (Group A, Group B, Group E) as the dependent variable, and the selected spirometry and EIT metrics as independent predictors. *Post hoc* model interpretation was performed using SHAP (Shapley Additive exPlanations) values. The average absolute SHAP values (mean |SHAP|) were calculated to rank the global importance of each feature. A Sankey diagram was employed to visualize the directional influence and flow of features on prediction outcomes.

### 2.5 Statistical analysis

Statistical analyses were performed using IBM SPSS Statistics for Windows (version 25.0; IBM Corp., Armonk, NY, United States) and R software version 4.4.3. Data wrangling was performed using dplyr (version 1.1.4), statistical analyses were conducted using rstatix (version 0.7.2), data visualization was created with ggplot2 (version 2.4.0), and regression model building and training were carried out using caret (version 7.0–1). Participants were classified into ABE groups (Group A, B, or E) according to the GOLD 2024 guidelines (2), based on collected clinical and functional data.

For continuous variables, normality was assessed prior to analysis. Normally distributed variables are presented as mean ± standard deviation and compared using one-way ANOVA. Non-normally distributed variables are expressed as median (interquartile range, 25th–75th percentile) and analyzed using the Kruskal–Wallis test, with *post hoc* comparisons performed using Dunn’s test where appropriate. To further minimize the impact of outliers on subsequent analyses, Mahalanobis distance was employed to identify and remove outliers. Outliers were determined using a chi-square distribution with degrees of freedom equal to the number of feature dimensions. A stringent confidence level of 99.9% (corresponding to *p* < 0.001) was applied. Categorical variables, reported as frequencies and percentages, were compared using the chi-square test or Fisher’s exact test. Correlations between SPIROMETRY parameters, EIT metrics, and CAT scores were assessed using Spearman’s rank correlation. A *p* < 0.05 was considered statistically significant.

## 3 Results

**FIGURE 1:**
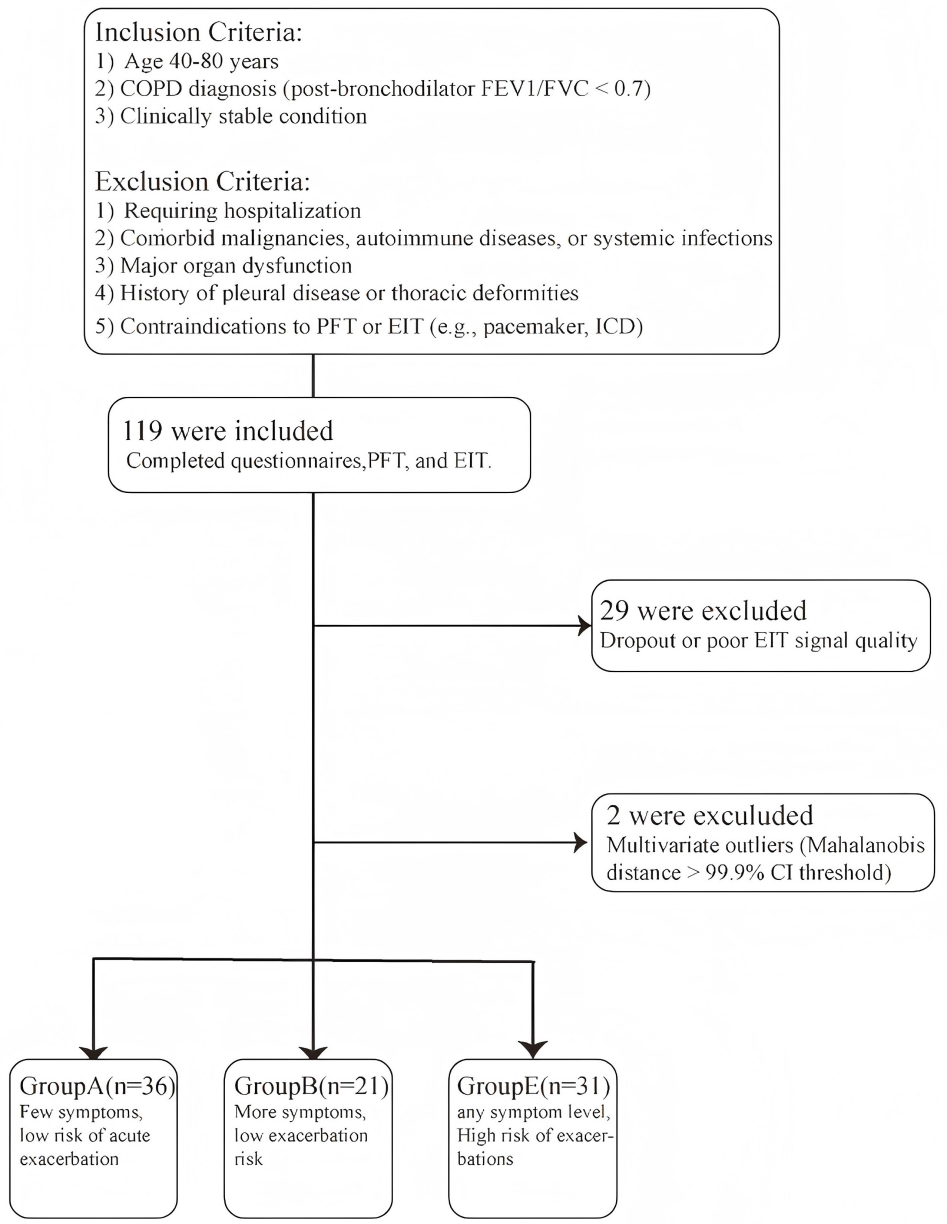
Enrollment flowchart.

**FIGURE 2:**
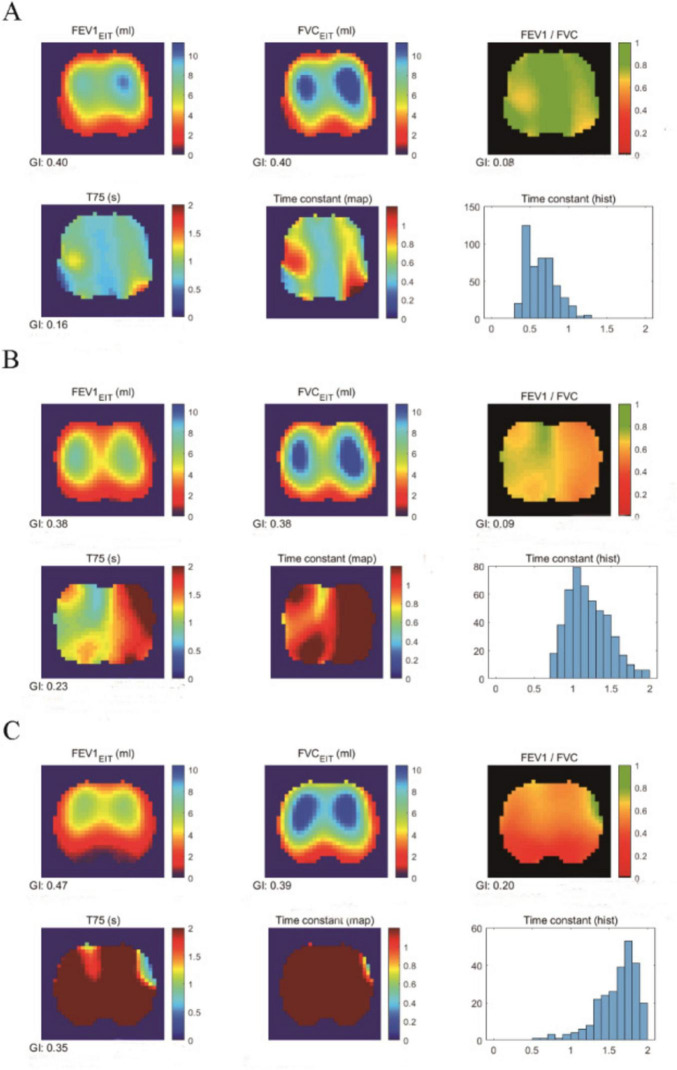
EIT images of COPD patients in Groups A, B, and E. **(A)** EIT imaging of Group A, **(B)** EIT imaging of Group B, and **(C)** EIT imaging of Group E, illustrating regional lung function measurements derived from electrical impedance tomography (EIT) in patients with COPD. These measurements include 1-s forced expiratory volume (FEV_1_), forced vital capacity (FVC), FEV_1_/FVC ratio, and the time required to exhale to 75% of FVC. These regional functional parameters were used to calculate the global inhomogeneity (GI) index for statistical analysis.

In this study, a total of 119 participants successfully completed baseline data collection, questionnaires, spirometry and EIT examination. The overall study enrollment is illustrated in Figure 1. Twenty-nine samples with poor EIT signal quality were excluded. The Mahalanobis distance suggested that two samples were outliers. The final analytical cohort comprised 88 participants. The final sample distribution across the ABE groups was as follows: 36 in Group A, 21 in Group B, and 31 in Group E. Figure 2 demonstrated the regional spatial and temporal differences among the groups.

### 3.1 Baseline characteristics

The baseline characteristics of participants in each group are summarized in Table 1. No significant differences were observed in most of the parameters among the three groups. However, a statistically significant difference was observed in the prevalence of long-term oxygen therapy among the three groups (*P* = 0.031). Group B had the highest proportion of participants receiving long-term oxygen therapy, which may be attributed to the higher symptom burden in this group and the corresponding therapeutic need for oxygen supplementation.

**TABLE 1:** Baseline characteristics of study participants.

Variables	Group A (n = 36)	Group B (n = 21)	Group E (n = 31)	*P*-value
**Sex, n (%)**				
Female	5 (13.8)	6 (28.8)	6 (19.4)	0.400
Male	31 (86.1)	15 (71.4)	25 (80.6)	
Age, years	59.53 ± 8.45	61.81 ± 10.51	59.00 ± 11.64	0.598
Height, cm	170.28 ± 8.09	167.10 ± 5.69	167.87 ± 7.98	0.241
Weight, kg	69.50 (60.00, 74.00)	63.00 (55.00, 75.00)	65.00 (56.50, 70.50)	0.364
**BMI, n(%)**				
Underweight	2 (5.5)	2 (9.5)	3 (9.7)	0.230
Normal weight	22 (61.1)	8 (38.1)	18 (58.1)	
Overweight	11 (30.6)	8 (38.1)	10 (32.3)	
Obesity	1 (2.8)	3 (14.3)	0	
**Smoking index classification, n(%)**				
Non-smoker	10 (27.8)	7 (33.3)	10 (32.3)	0.377
Light smoker	3 (8.3)	3 (14.3)	3 (9.6)	
Moderate smoker	5 (13.9)	3 (14.3)	10 (32.3)	
Heavy smoker	18 (50.0)	8 (38.1)	8 (25.8)	
**Exposure history of toxic and harmful particles, n(%)**				
No	28 (77.8)	19 (90.5)	24 (77.4)	0.428
Yes	8 (22.2)	2 (9.5)	7 (22.6)	
**Exposure history of organic dyes, n (%)**				
No	34 (94.5)	20 (95.2)	30 (96.8)	0.900
Yes	2 (5.5)	1 (4.8)	1 (3.2)	
**Long-term oxygen therapy status, n (%)**				
No	35 (97.2)	16 (76.2)	24 (77.4)	0.031
Yes	1 (2.8)	5 (23.8)	7 (22.6)	

BMI categories were defined according to the Chinese reference standards: underweight (BMI < 18.5), normal weight (BMI 18.5–23.9), overweight (BMI 24–27.9), and obesity (BMI ≥ 28). The smoking index was calculated as the number of cigarettes smoked per day multiplied by the number of smoking years, and stratified into three risk levels: low risk (< 200), moderate risk (200–400), and high risk (> 400).

### 3.2 Intergroup differences in clinical and EIT metrics

**FIGURE 3:**
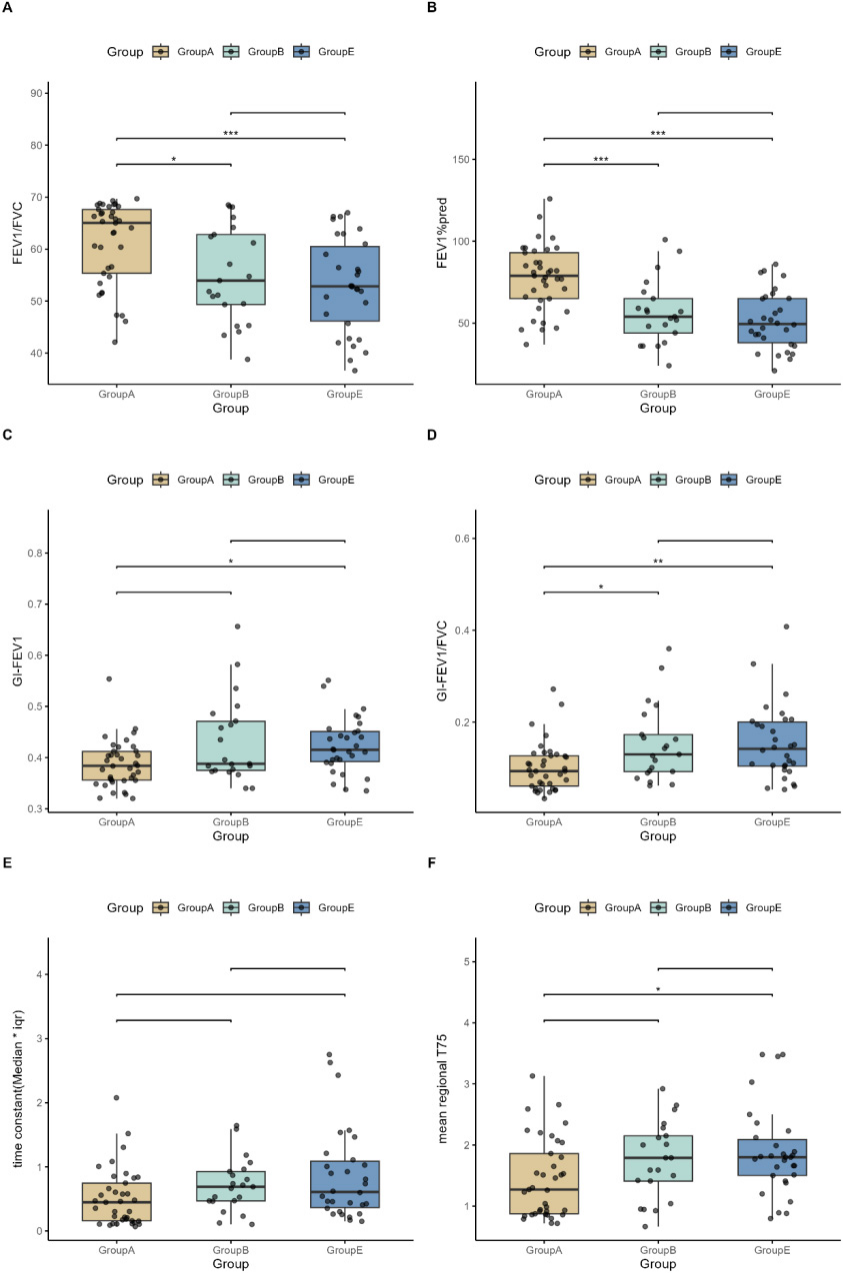
Pairwise comparisons of study indicators among the A, B, and E groups. **(A–F)** Illustrate pairwise comparisons of the following key variables between Groups A, B, and E: FEV_1_/FVC (%), FEV_1_% pred, GI-FEV_1_, GI-FEV_1_/FVC, time constant (Median*iqr), and mean regional T75. Statistical significance is indicated as follows: *P < 0.05, **P < 0.01, and ***P < 0.001.

Table 2 and Figure 3 present the intergroup differences in FEV_1_%pred, FEV_1_/FVC, GI-FEV_1_, GI-FEV_1_/FVC, and τ among the A, B, and E groups. Following *post hoc* multiple comparisons, Group A demonstrated significantly higher values in FEV_1_%pred (*P* < 0.001) and FEV_1_/FVC (*P* < 0.05) compared to Group B and Group E, respectively. Similarly, the GI-FEV_1_/FVC value in Group A was significantly lower than that in Group B and Group E (both *P* < 0.05).

**TABLE 2:** Differences in related indicators among the A, B, and E groups.

Parameters	Group A (n = 36)	Group B (n = 21)	Group E (n = 31)	*P*-value
FEV_1_%pred	77.67 ± 20.40	56.62 ± 19.63	50.39 ± 16.45	<0.001
FEV_1_/FVC	65.17 (55.02, 67.90)	53.95 (49.33, 62.80)	52.76 (44.36, 60.00)	<0.001
GI-FEV_1_	0.38 (0.36, 0.41)	0.39 (0.36, 0.47)	0.42 (0.39, 0.46)	0.004
GI-FEV_1_/FVC	0.09 (0.06, 0.13)	0.13 (0.09, 0.17)	0.15 (0.11, 0.21)	0.001
τ	0.40 (0.16, 0.79)	0.69 (0.47, 0.93)	0.62 (0.38, 1.16)	0.017
Mean regional T75	1.27 (0.87, 1.95)	1.79 (1.41, 2.15)	1.81 (1.58, 2.18)	0.009

*P*-values denote statistical significance across the three groups. Normally distributed variables were analyzed using one-way ANOVA, and non-normally distributed variables were compared using the Kruskal-Wallis test.

### 3.3 Correlation between CAT scores and study metrics

**FIGURE 4:**
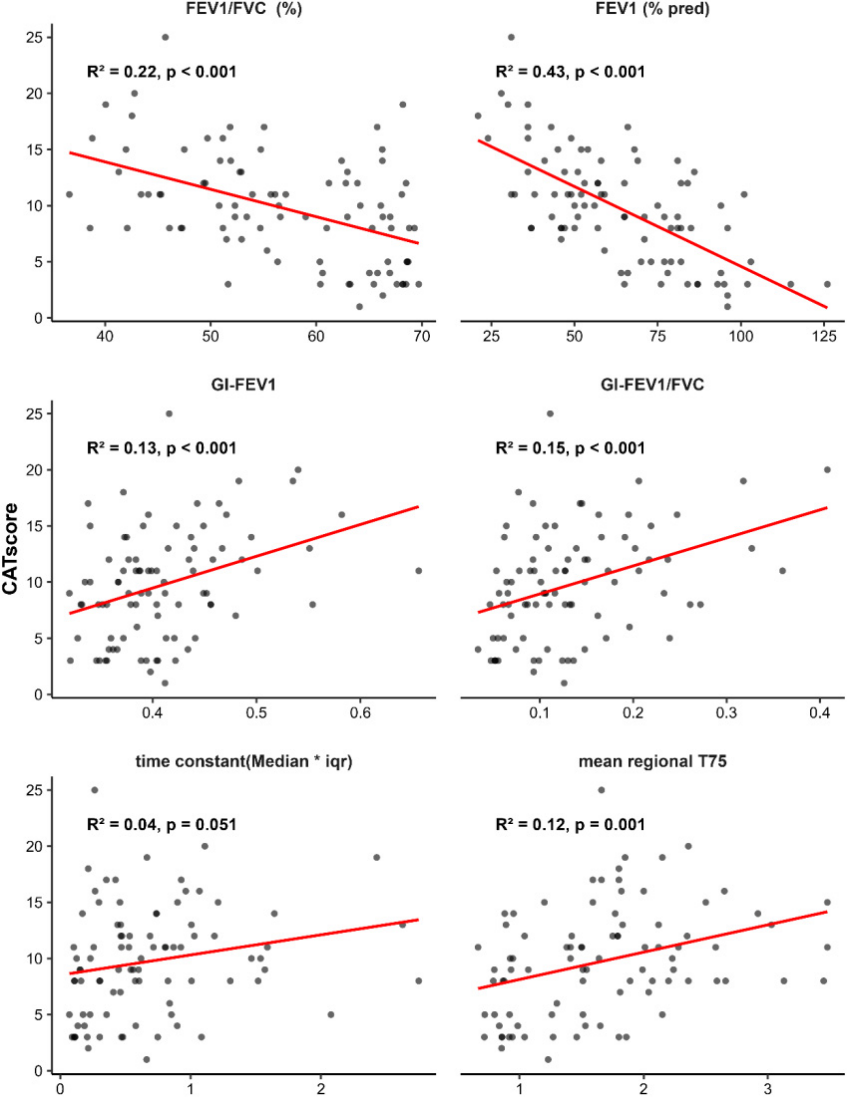
Correlation analysis between CAT score and study indicators. The correlation analysis between the COPD Assessment Test (CAT) score and the following key variables: FEV_1_/FVC (%), FEV_1_% pred, GI-FEV_1_, GI-FEV_1_/FVC, time constant (Median*iqr), and mean regional T75. The coefficient of determination (R^2^) reflects the strength of the correlation, and the *P*-value indicates statistical significance.

Further correlation analysis was conducted to evaluate the associations between CAT scores and the study’s key metrics across all groups. The results, illustrated in Figure 4, demonstrated that all examined indicators showed statistically significant correlations with CAT scores. Among these, FEV_1_%pred exhibited the strongest correlation (*R*^2^ = 0.43), followed by the FEV_1_/FVC (*R*^2^ = 0.22).

### 3.4 Principal component analysis

**FIGURE 5:**
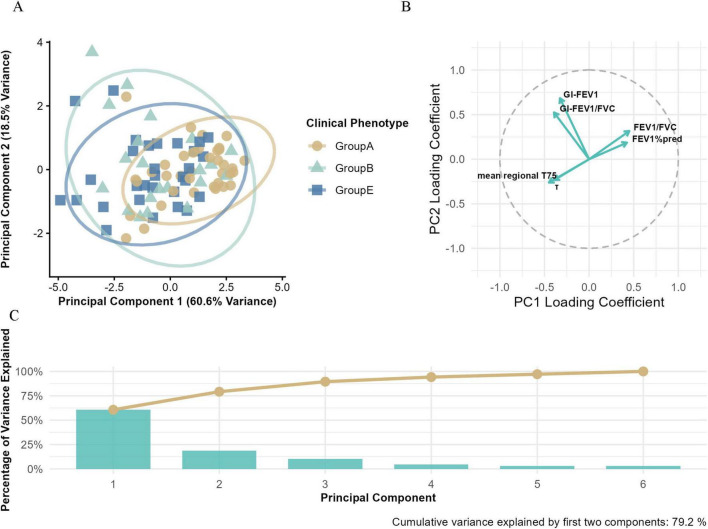
Principal component analysis (PCA) of Groups A, B, and E. **(A)** The PCA based on electrical impedance tomography (EIT) parameters and conventional pulmonary function metrics, illustrating the multidimensional separation of clinical phenotypes. **(B)** The loading plot that visualizes the contribution of variables to principal components (PCs), with the horizontal axis representing PC1 loading coefficients and the vertical axis representing PC2 loading coefficients. **(C)** The variance explained by principal components, where blue bars indicate the variance explained by individual PCs and the orange line shows the cumulative variance explained.

As shown in Figure 5, the first two principal components (PC1 and PC2) explained 79.1% of total variance (PC1: 60.6%; PC2: 18.5%).

Clear clustering was observed along PC1. Group E patients were mainly located in the negative quadrant, while Groups A and B shifted progressively toward the positive side, suggesting PC1 may reflect a continuum of disease severity.

Within the PC space, Group A showed tight clustering in the positive PC1 quadrant, indicating high phenotypic homogeneity. In contrast, Group E displayed broader dispersion along PC1, implying greater physiological heterogeneity.

Factor loadings revealed that PC1 was primarily driven by FEV_1_%pred (0.42), FEV_1_/FVC (0.42), mean regional T75 (–0.45), and τ (–0.39), representing a combination of airflow limitation and ventilation impairment. PC2 was more strongly associated with regional ventilation heterogeneity metrics, including GI-FEV_1_ (0.68) and GI-FEV_1_/FVC (0.52) mean regional T75(−0.26), τ(−0.23), suggesting spatial and temporal variability in lung damage.

### 3.5 Multinomial logistic regression analysis

Model performance was comprehensively evaluated using multiple visualization methods, and the decision-making mechanism was further interpreted.

#### 3.5.1 Overall model performance

**FIGURE 6:**
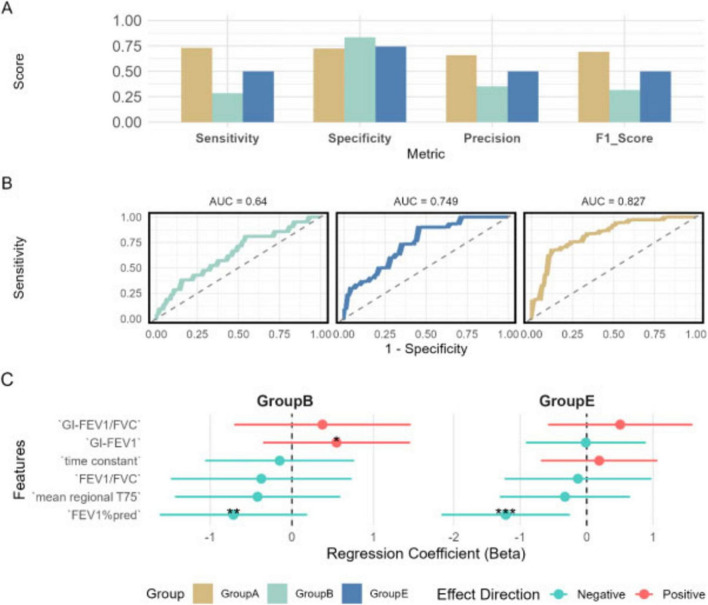
Comprehensive evaluation of the multinomial logistic regression model performance. **(A)** Model performance metrics, displaying the F1 score, sensitivity, precision, and specificity for each phenotype. **(B)** Multiclass receiver operating characteristic (ROC) curves, illustrating the model’s discriminative ability for each phenotype, with area under the curve (AUC) values indicating classification performance. **(C)** The global average effects of features in both Group B and Group E models.

Model performance was assessed using radar plots and ROC curves (Figure 6). Figure 6A indicated varying discriminative abilities across subgroups, with the best performance for Group A, followed by Group E, while discrimination for Group B was relatively limited.

This trend was further supported by multiclass receiver operating characteristic (ROC) curves constructed using a one-vs.-one strategy. The corresponding areas under the curve (AUCs) were: Group A 0.827, Group E 0.749, Group B 0.640 (Figure 6B).

Figure 6C displays the regression coefficients of various features in Group B and Group E, reflecting their global effects. Among these, FEV_1_%pred exhibits a significant negative effect in both groups. Besides FEV_1_%pred, features such as FEV_1_/FVC and mean regional T75 also show negative effects in both models. In contrast, GI-FEV_1_/FVC demonstrates a positive effect in both groups. Notably, GI-FEV_1_ and τ exhibit distinctly divergent effects between the two models. Specifically, GI-FEV_1_ shows a positive effect in Group B but a negative effect in Group E. Conversely, the effect of τ is positive in Group E but negative in Group B.

#### 3.5.2 Feature importance and directional impact analysis

**FIGURE 7:**
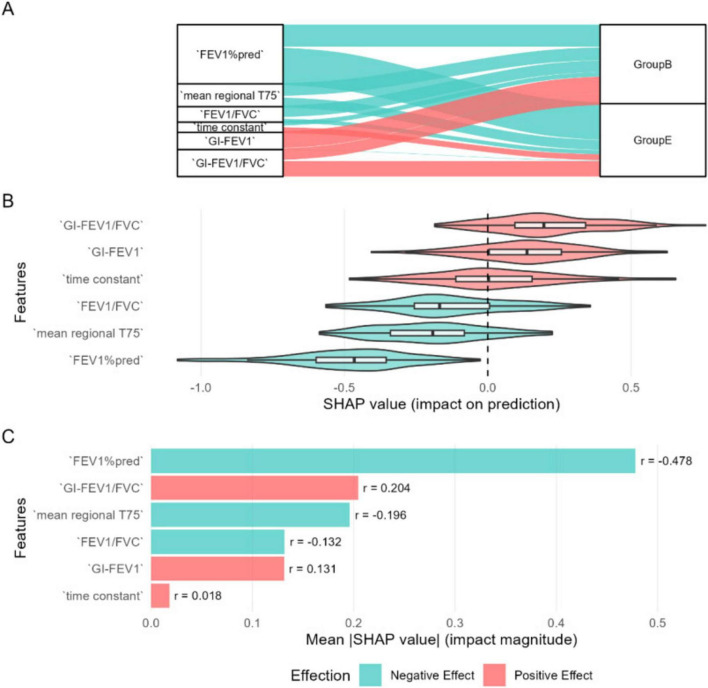
Interpretability analysis of the multinomial logistic regression model. **(A)** Sankey diagram illustrating feature-class relationships (blue: negative influence, red: positive influence). **(B)** SHAP value distribution plot, where the r value represents the correlation between each feature and its corresponding SHAP values. **(C)** Feature importance ranking based on mean |SHAP|.

Figure 7 illustrates the feature importance ranking and directional impact analysis of the multinomial logistic regression model in classification decisions. The SHAP summary plot (Figure 7A) visually demonstrates the directional relationships between key features and predicted classes. FEV_1_%pred, as the strongest predictor, exerts a dominant influence on classifications into Group B, whereas the EIT-derived parameters GI-FEV_1_/FVC and τ primarily drive classifications into Group E.

The feature importance ranking based on mean |SHAP| values (Figure 7B) shows that FEV_1_%pred is the most critical predictor for distinguishing between Group B and Group E (|SHAP| = 0.477), with a contribution significantly higher than other variables. The EIT-derived parameters GI-FEV_1_/FVC (|SHAP| = 0.203) and mean regional T75 (|SHAP| = 0.189) rank as the second and third most important features, respectively, while the time heterogeneity indicator τ has the weakest predictive contribution (|SHAP| = 0.011).

The SHAP value distribution (Figure 7C) further clarifies the directional influence of each feature on prediction outcomes: the SHAP values of FEV_1_%pred are predominantly concentrated in the negative range (*r* = −0.477), indicating that higher values of this metric increase the likelihood of the model classifying a patient into Group B. In contrast, the EIT-derived parameters GI-FEV_1_/FVC (*r* = 0.203) and GI-FEV_1_ (*r* = 0.141) show SHAP values primarily in the positive range, meaning higher values of these indicators make the model more likely to assign the patient to Group E. For mean regional T75 (*r* = −0.196), higher values also tend to drive the model toward classifying the patient as Group B. However, τ, which is highly correlated with mean regional T75 but reflects temporal heterogeneity, shows the opposite trend, favoring classification into Group E.

## 4 Discussion

In this study, we explored the regional ventilation patterns across the ABE subgroups using EIT, with the aim of identifying significant differences in ventilation patterns among the groups. Furthermore, we tested various predictive models combining EIT-derived metrics and spirometry parameters to better distinguish the ABE subgroups. The results demonstrated significant statistical differences in the studied indicators across the three groups. However, pairwise comparisons revealed no significant differences between Groups B and E, suggesting potential overlaps in their pathophysiological characteristics. In the constructed multinomial logistic regression model, the pulmonary function parameter FEV_1_%pred and the EIT parameter GI-FEV_1_/FVC played pivotal roles in group discrimination. Further exploration in this direction could enhance our understanding of how EIT metrics contribute to identifying Group E.

Compared to conventional pulmonary function testing (e.g., spirometry)—the current gold standard for diagnosing COPD and monitoring disease progression–EIT is unable to provide absolute values of the pulmonary function parameters, but the information regarding regional ventilation distribution (18). In this study, we analyzed correlations between the six pulmonary function and EIT indicators and CAT scores. All indicators exhibited statistically significant associations with CAT scores. As a subjective measure reflecting COPD severity, CAT scores showed a strong negative correlation with FEV_1_%pred (R^2^ = 0.43) and a robust association with the EIT-derived regional ventilation heterogeneity metric GI-FEV_1_/FVC (*R*^2^ = 0.15). This finding was different from a previous study (7). It might be due to the questionnaires used in the studies. St. George’s Respiratory Questionnaire was used in the previous study, which is more thorough but time consuming. CAT is simpler. Prospective study should be conducted to confirm the hypothesis.

PCA analysis revealed two core dimensions of COPD heterogeneity: PC1 (60.6% variance), integrating the severity of airflow limitation and gas trapping, aligned with the gradient of GOLD grouping severity, consistent with the phenotype gradient described by Han et al. (11); PC2 (18.5% variance) primarily captured regional ventilation heterogeneity. The multi-parameter loading pattern indicated that traditional pulmonary function metrics (e.g., FEV_1_%pred) and EIT parameters (e.g., GI index, τ) contributed to phenotype differentiation from both global and local perspectives, collectively establishing a multidimensional assessment framework for COPD.

As a high-risk COPD subgroup, promptly identifying and intervening in Group E patients is clinically crucial for halting disease progression, reducing exacerbation frequency, and improving prognosis. The differential analysis revealed that, compared to Group A, Groups B and E exhibited significant regional ventilation dysfunction, characterized by spatial distribution unevenness, increased temporal heterogeneity, and exacerbated gas trapping (all intergroup *P* < 0.05). Although no significant mean differences were observed in the six indicators between Groups B and E (*P* > 0.05)—potentially attributable to the limited sample size and shared pathological and physiological traits—the PCA results demonstrated that Group E patients were predominantly distributed in the negative quadrant of PC1, indicating more severe overall lung function impairment and prominent gas trapping. This aligns closely with the core pathophysiological features of Group E, which are centered on pulmonary parenchymal destruction and gas trapping (12). In contrast, Group B patients showed a narrower distribution along PC1, suggesting their pathological changes are more focused on airway inflammation rather than emphysematous alterations. Additionally, Group B and E patients exhibited broader dispersion along PC2, significantly higher than Groups A, reflecting pronounced regional ventilation heterogeneity within the groups. PCA factor loading analysis further confirmed that PC2 was most strongly associated with the regional ventilation heterogeneity indicator GI-FEV_1_, substantiating the presence of significant spatial ventilation distribution unevenness in Group B and E. This finding suggests that Group B and Group E may encompass multiple subtypes with distinct ventilatory function characteristics, which cannot be simply defined by chronic bronchitis and emphysema alone, warranting further subgroup analysis using imaging or biomarkers to enable more precise personalized treatment.

Although the established multinomial logistic regression model in this study provided good interpretability, its discriminatory performance for Groups B and E remained suboptimal (AUC = 0.640 and 0.749, respectively). We speculated that several factors may influence the model’s recognition ability: (1) pathological and physiological overlaps between Groups B and E, where some patients may exhibit features of both phenotypes; (2) the current feature set may not fully capture the intrinsic differences between the two groups, potentially necessitating the inclusion of inflammatory markers or imaging parameters; and (3) the relatively small sample size, which may introduce bias and affect the model’s accuracy and stability.

In the SHAP interpretability analysis, we further elucidated the predictive efficacy and decision-making mechanisms of pulmonary function indicators and EIT parameters in distinguishing COPD clinical phenotypes. The results revealed that the traditional pulmonary function metric FEV_1_%pred was the most influential predictor for differentiating Groups B and E (|SHAP| = 0.477), with a contribution significantly higher than other variables. Additionally, EIT-derived parameters—mean regional T75 (|SHAP| = 0.189) and GI-FEV_1_/FVC (|SHAP| = 0.203)—ranked as the second and third most important features, respectively, playing a critical role in distinguishing Groups B and E. This suggests potential differences in regional ventilation heterogeneity and ventilation delay between the two groups, consistent with their underlying pathophysiological disparities (7, 12). Notably, although τ and mean regional T75 exhibited consistency, their influences on Groups B and E differed. This indicates that the τ is more sensitive to pulmonary airflow heterogeneity and may effectively identify specific subgroups with severe gas trapping. Furthermore, PCA results suggested the presence of distinct subgroups within Group E, which contributed to the low global average contribution of the τ (|SHAP| = 0.011). However, we suspected that increasing the sample size or conducting further subgroup analysis within Group E could enable the τ to serve as a valuable indicator for identifying subgroups with severe gas trapping, which was not explored in the current study.

This study has several limitations. First, the relatively small sample size may not only affect model stability and generalizability but also reduce the accuracy of feature selection, limiting the exploration of the classification potential of the included indicators. Second, due to the limited sample size and to avoid model overfitting, the current feature set did not include well-established COPD-related diagnostic indicators such as inflammatory markers and imaging parameters, potentially omitting key discriminative information and depriving the model of critical factors. Third, the cross-sectional design precludes the assessment of parameter changes over time and their relationship with long-term prognosis. This restricts an in-depth exploration of the dynamic evolution patterns and potential causal associations of EIT indicators across ABE groups, leading to an unclear evaluation of the model’s capability for dynamic monitoring of COPD and its potential clinical applications. Consequently, the true feature changes during disease progression cannot be fully reflected. Fourth, the mucus plugs, on one hand may influence the EIT and spirometry results, on the other hand, may be a risk factor for exacerbations. Depending on the degree and the time of presence (e.g. could presence after CAT but before spirometry), it adds bias for the analysis and interpretations.

## 5 Conclusion

COPD phenotypes showed differences in global and regional flow limitations. The combination of global and regional information helped with distinguishing phenotypes.

## Data Availability

The original contributions presented in the study are included in the article/supplementary material, further inquiries can be directed to the corresponding authors.
